# The chondroitin/dermatan sulfate synthesizing and modifying enzymes in laryngeal cancer: Expressional and epigenetic studies

**DOI:** 10.1186/1758-3284-2-27

**Published:** 2010-10-07

**Authors:** Dimitrios Kalathas, Irene-Eva Triantaphyllidou, Nicholas S Mastronikolis, Panos D Goumas, Thoedore A Papadas, Gabriel Tsiropoulos, Demitrios H Vynios

**Affiliations:** 1 Department of Chemistry, Laboratory of Biochemistry - Section of Organic Chemistry and Natural Products, Karatheodori str., University of Patras, Patras, 26500, Greece; 2 Department of Otorhinolaryngology-Head and Neck Surgery, University Hospital of Patras, Hippokrates str., Patras, 26500, Greece

## Abstract

**Background:**

Significant biochemical changes are observed in glycosaminoglycans in squamous cell laryngeal carcinoma. The most characteristics are in chondroitin/dermatan sulfate fine structure and proportion, which might be due to differential expression of the enzymes involved in their biosynthesis. The aim of the present work was the investigation in expressional and epigenetic level of the enzymes involved in chondroitin/dermatan sulfate biosynthesis in laryngeal cancer.

**Methods:**

Tissues subjected to total RNA and DNA isolation, and protein extraction. The techniques used in this study were RT-PCR analysis, western blotting and methylation specific PCR.

**Results:**

We identified that many enzymes were expressed in the cancerous specimens intensively. Dermatan sulfate epimerase was expressed exclusively in the cancerous parts and in minor amounts in healthy tissues; in the macroscopically normal samples it was not detected. Furthermore, chondroitin synthase I and chondroitin polymerizing factor were strongly expressed in the cancerous parts compared to the corresponding normal tissues. Sulfotransferases, like chondroitin 6 sulfotransferase 3, were highly expressed mainly in healthy specimens.

**Conclusions:**

The study of the various chondroitin/dermatan synthesizing enzymes revealed that they were differentially expressed in cancer, in human laryngeal cartilage, leading to specific chondroitin/dermatan structures which contributed to proteoglycan formation with specific features. The expression of the examined enzymes correlated with the glycosaminoglycan profile observed in previous studies.

## Background

The chondroitin/dermatan sulfate fine chemical structure is altered in laryngeal carcinomas [1,2] as well as in most cancers [3-5]. In healthy larynx, chondroitin/dermatan sulfate (C-6 and C-4 sulfated) in the cartilaginous parts is present in greater amounts compared to cancer. Moreover, the decrease in cancer is more abrupt in C-6 sulfation; C-4 sulfation is diminished gradually to the advanced stages of cancer. These alterations may be due to differential biosynthesis of core protein precursors, to differences in the substrates pool, and to differential expression of the enzymes involved in chondroitin/dermatan sulfate biosynthesis.

The chondroitin and dermatan sulfate synthesizing and modifying enzymes are characterized [6-12]. Chondroitin synthases (CHSY1, CHSY2 (CHPF) and CHSY3) and chondroitin sulfate glucuronyltransferase (CSGlcA-T) polymerize the glycosaminoglycan chains. The polymer modifying enzymes include sulfotransferases (C4ST1, D4ST1, C4ST2, C4ST3 and CHST3) and dermatan sulfate epimerase (DSE). In a previous study, we demonstrated that CHST3 and D4ST1 were expressed differentially in colorectal cancer [4], thus leading to increased C-6 sulfation in cancer compared to healthy tissues and decreased C-4 sulfation in late stages.

The purpose of the present study was to examine the expression of the various chondroitin/dermatan sulfate synthesizing and modifying enzymes in laryngeal cartilage in healthy, macroscopically normal and cancerous specimens by RT-PCR analysis and western blotting. Furthermore, methylation specific PCR (MSP) was used to find out if DNA methylation is a regulative mechanism of their expression in laryngeal cancer.

## Methods

### Chemicals and biologic material

Each cartilaginous laryngeal tissue specimen (28 samples, 60 ± 11 y) of male patients with advanced cancer stage (stages III and IV) was separated into normal and cancerous part. Specimens of healthy donors (4 samples, aged 50 ± 14 y) were also included in the study. The study design was approved by the Ethical Committee of the University Hospital of Patras and was in accordance with the Helsinki Declaration of 1975, as revised in 1983.

Phosphate buffer saline (PBS) and phenylmethylsulfonyl fluoride (PMSF) were obtained from Serva (Darmstadt, Germany). Benzamidine hydrochloride, ε-amino-n-caproic acid, Triton X-100, N-ethylmaleimide and disodium EDTA were obtained from Sigma. RNA extraction kit (Nucleospin RNA II) was from Macherey-Nagel (Düren, Germany). PrimeScript™one step RT-PCR kit and 100 bp DNA ladder were obtained from TakaRa BIO INC (Ōtsu, Japan). Goat antibodies against CHSY1 (N-13), CHSY2 (E-19), CHSY3 (C-17) and C4ST1 (N-18) were purchased from Santa Cruz Biotechnology, INC (Santa Cruz, USA). Rabbit anti-goat IgG horseradish peroxidase conjugated was from Chemicon (California, USA). DNeasy Blood & Tissue Kit, RNAse A, EpiTect^® ^Bisulfite kit and HotStarTaq DNA Polymerase were from Qiagen. RNA extraction kit (Nucleospin RNA II) was from Macherey-Nagel (Düren, Germany). ECL Western Blotting Substrate was from Pierce biotechnology (Rockford, USA). The gene specific primers were purchased by Lieferschein (Germany). All other chemicals used throughout the study were of the highest available grade.

### RNA extraction and RT-PCR

RNA extraction, RT-PCR analysis and agarose gel electrophoresis were performed essentially as described elsewhere [4]. The specimens were pulverized in liquid nitrogen and subjected to total RNA exctraction using the Nucleospin extraction kit and treated with RNase-free DNase to remove contaminating genomic DNA. The primers used (illustrated in table 1) were designed using a free software (PerlPrimer v1.1.14). Takara one step RT-PCR kit was used to perform the analysis. The conditions of RT-PCR were as previously [4]. RT-PCR products were separated by gel electrophoresis on 2% w/v agarose gel containing SYBR Gold stain to visualize the bands under UV. The gels were then scanned and the bands were analyzed densitometrically. Quantitative differences between cDNA samples were normalized by including GAPDH in all experiments.

**Table 1:** Nucleotide sequence of the primers used in RT-PCR experiments

Type of primer	Nucleotide sequence (5-3)
CHSY1-F	AGTGTGTCTGGTCTTATGAGATGCA
CHSY1-R	AGCTGTGGAGCCTGTACTGGTAG
CHPF-F	GTCAGGACCCGCTACATCAG
CHPF-R	CTCTCCGCCGATGAAGTCCT
CHSY3-F	CGATGTCTACATCAAAGGTGACAAA
CHSY3-R	GCTGGAAGTGGTTGAAAGAAGG
CSGlcAT-F	AGAACAACTGCAGGCTCAGATCC
CSGlcAT-R	AGAGTGTGGTGTGAAAGGAGCAG
CHST3-F	CATATCAAGGGTCTCAGACAAGC
CHST3-R	GTACAGGTCGCACAGGAAGAG
C4ST1-F	AAGTATGTTGCACCCAGTCATGC
C4ST1-R	TTCAAGCGGTTGTTGATTTCTGG
D4ST1-F	ACTGGAAGCGGGTGATGAAGG
D4ST1-R	AAATCGGACGTGAGGTGGTGC
DSE-F	TGGTTGGTGAAAGATGCTCCT
DSE-R	GTCCTTTGAAACCCTGGCAG
GAPDH-F	TCAAGATCATCAGCAATGCCTCC
GAPDH-R	AGTGAGCTTCCCGTTCAGC

### DNA isolation and MSP

Tissue specimens were treated for DNA isolation with DNeasy Blood & Tissue Kit. For complete bisulfite conversion and cleanup of DNA for methylation analysis the Epitect^® ^Bisulfite kit was used. The processes were performed as follows: The tissue specimens were pulverized in liquid nitrogen and then incubated in proteinase K solution at 56°C overnight. Then RNase A was added to remove any contaminating RNA followed by a short incubation (2 min) at 25°C. The isolated DNA was used for the conversion of unmethylated C to U. Eight hundred ng of total DNA was used to be converted by the bisulfite reaction in a thermal cycler. The thermal cycler conditions for the conversion were 3 successive steps of denaturation and incubation. Each denaturation step took place at 99°C for 5 min and each incubation step at 60°C. The first incubation step was for 25 min, the second for 1 h and 25 min and the third for 2 h and 55 min. One hundred ng of DNA was used for MSP. The primers (illustrated in table 2) were constructed manually. The conditions for MSP were as follows: Taq polymerase was activated at 95°C for 15 min, followed by 35 amplification cycles and 10 min at 72°C. Each cycle consisted of 3 steps which were as follows: For DSE, 94°C for 30 s, 60°C for 30 s and 72°C for 1 min. For C4ST1, 94°C for 30 s, 52.5°C for 45 s and 72°C for 1 min. Finally, for D4ST1, 94°C for 30 s, 58.5°C for 35 s and 72°C for 1 min. PCR products were separated by gel electrophoresis on 3% w/v agarose gel containing SYBR Gold stain to visualize the bands under UV. The gels were then scanned.

**Table 2:** Nucleotide sequence of the primers used in the MSP experiments.

Type of primer	Nucleotide sequence (5-3)
DSE-UF	**GTGG**AGGTGATGTTGGAGAGAAT
DSE-MF	AGGCGACGTCGGAGAGAAC
DSE-UR	**AAAC**TACCCACCCAAAACTCCCA
DSE-MR	TACCCGCCCGAAACTCCCG
D4ST-UF	**GG**GTGGAGAGTGGTTGGGT
D4ST-MF	GTGGAGAGCGGTCGGGC
D4ST-UR	**AATAA**CCAACTCCCAAACTACAACA
D4ST-MR	CCGACTCCCGAACTACGACG
C4ST-UF	TTAGATGGTGGTTGAGTTT
C4ST-MF	TTAGACGGCGGTCGAGTTC
C4ST-UR	**AAAATATA**ATCACATCCAACTATTAACCA
C4ST-MR	ATCGCGTCCAACTATTAACCG

The longer segments of the unmethylated primers are in boldface type and differences between methylated/modified and ummethylated/modified sequence are underlined underlined

### Protein extraction-Western blotting

Parts of healthy, macroscopically normal and cancerous tissues were used for the detection of chondroitin synthases and chondroitin sulfate 4-sulfotransferase. Each specimen was finely diced and the macromolecules contained were sequentially extracted for 3X24 h periods at 4°C in the darkness with PBS (10 mM disodium phosphate, 0.14 M NaCl, pH 7.4), 4 M GdnHCl-0.05 M sodium acetate pH 5.8 and 4 M GdnHCl-0.05 M sodium acetate-1% Triton X-100 pH 5.8, using 10 vols of extraction buffer per g of tissue. A protease inhibitor cocktail was also included [13]. The samples were thereafter subjected to western blotting as described [14].

## Results

### Chondroitin synthases and glucuronyltransferase expression

**Figure 1:**
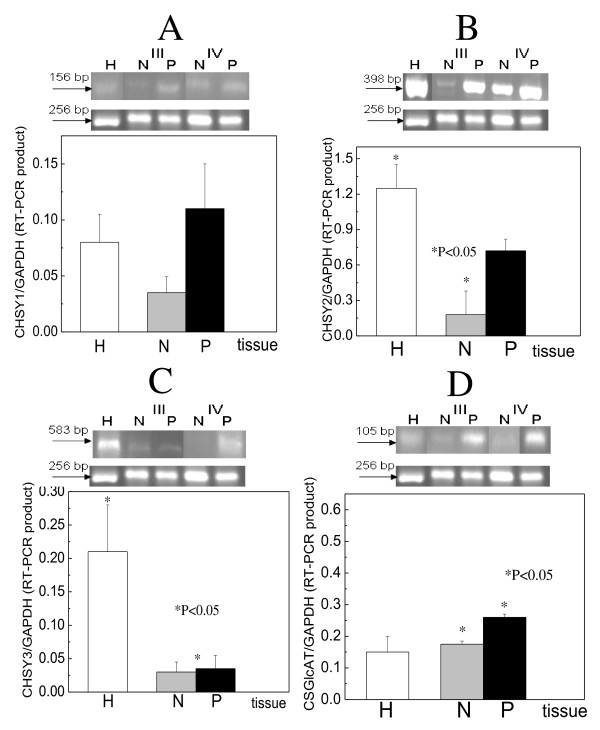
**RT-PCR analysis of the chondroitin/dermatan synthesizing enzymes**. A. CHSY1, B. CHPF, C. CHSY3 D. CSGlcA-T, in macroscopically normal (N) and pathological (P) specimens. White boxes, healthy; grey boxes, macroscopically normal; black boxes, cancerous

**Figure 2:**
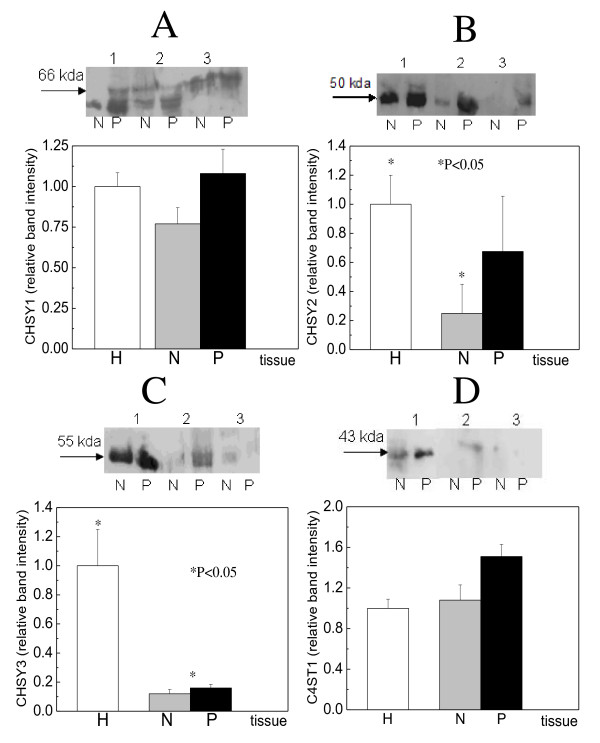
**Western blot analysis in the sequential extracts**. A. CHSY1 B. CHSY2 C. CHSY3 and D. C4ST1. 1. PBS 2. 4 M GdnHCl-0.05 M sodium acetate and 3. 4 M GdnHCl-0.05 M sodium acetate-1% Triton X-100. Five microlitres were used for PBS extracts and fifty microlitres for both others.

CHSY1 gene expression in healthy tissues was about to one twelfth of that of GAPDH (fig 1A). In pathologic samples, increased expression was detected compared to healthy, being 30% as indicated by RT-PCR experiments (fig. 1A), and 10% from western blotting (fig. 2A). The macroscopically normal samples expressed the enzyme to a smaller extent compared to pathologic ones, being about 30% as indicated by RT-PCR (fig. 1A). About similar results were obtained using western blotting, however the expression found was higher (70%) (fig. 2A).

CHSY2 gene was the chondroitin synthase with the highest expression in healthy tissues (1.25 times the GAPDH levels), indicating its great importance in chondroitin/dermatan polymerization. A very clear decrease of its expression was observed in patients' specimens. Its expression, in the pathologic samples, was about half than that of the healthy specimens (P < 0.05), as indicated by RT-PCR analysis (fig. 1B) and western blotting (fig. 2B). Moreover, the pathologic samples, compared to adjacent normal, expressed the enzyme 2 to 3 times higher.

The healthy specimens expressed CHSY3 gene to the one fifth compared to GAPDH gene. The specified enzyme was expressed about in equal amounts between macroscopically normal and pathologic samples, but very low compared to healthy. Its decrease in cancer was about 8 times (P < 0.05) as indicated by both RT-PCR analysis and western blotting (fig. 1C, 2C).

The expression of CSGlcA-T gene was 7 times lower than that of GAPDH gene in healthy tissues. Its expression was found to be increased in cancer in both normal and pathologic samples, as indicated from RT-PCR experiments (fig. 1D). The increase was more profound in pathologic samples, being 50% and statistically significant (P < 0.05).

### Chondroitin sulfotransferases expression

**Figure 3:**
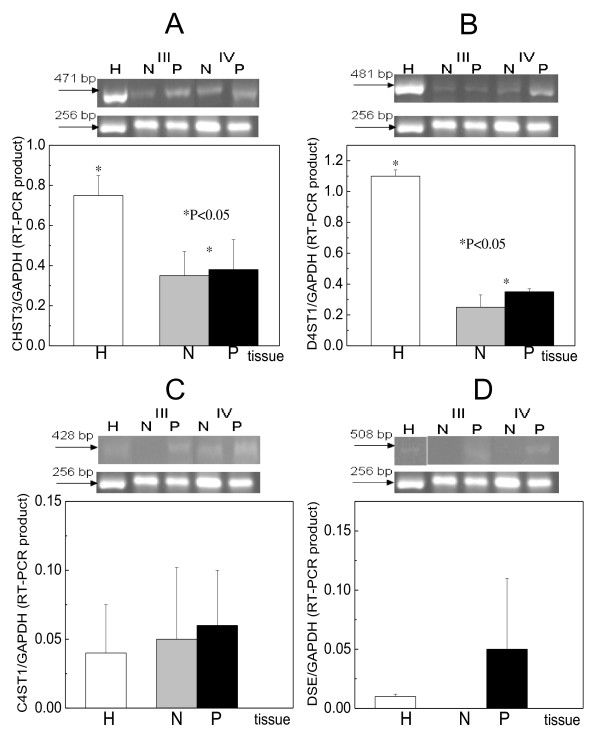
**RT-PCR analysis of chondroitin/dermatan modifying enzymes**. A. CHST3 B. D4ST1 C. C4ST1 and D. DSE. For details, see fig. 1.

CHST3 gene was expressed in healthy tissues about the same levels as the GAPDH gene (fig. 3A). Its expression was decreased in patients' specimens to 50% compared to healthy as indicated by RT-PCR analysis, being statistically significant (P < 0.05). Moreover, the expression was equal between the macroscopically normal and pathologic samples.

**Figure 4:**
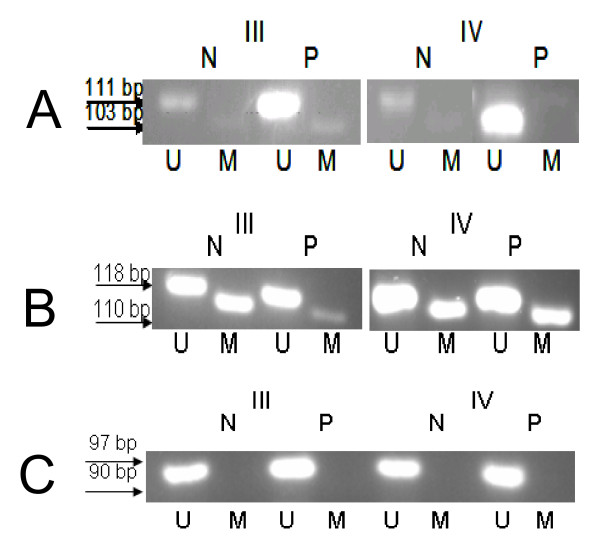
**Methylation specific analysis**. MSP for A. C4ST1, B. DSE and C. D4ST1 gene M and U, amplification using methylated and unmethylated sequence-specific primers, respectively.

C4ST1 gene expression was very low in healthy specimens (20 times lower compared to the GAPDH gene), and increased in patients' specimens as indicated by both RT-PCR and western blotting (fig. 3C,2D). Its expression seemed to be controlled via methylation of a CpG island, since hypomethylation of the gene was observed in the pathologic samples compared to the macroscopically normal samples (fig.4A).

D4ST1 gene was about equally expressed with the GAPDH gene and possessed its highest expression in the healthy tissues (fig. 3B). In cancer, its expression was decreased 4 to 5 times and it was about equal between normal and pathologic samples (fig. 3B). The CpG island near the promoter region was fully unmethylated therefore it did not affect the enzyme expression (fig. 4C).

### DSE expression

DSE gene was expressed in negligible amounts in the healthy tissues (more than 50 times lower expression than GAPDH gene, fig. 3D). DSE expression was not detected in the macroscopically normal samples, and the highest levels of it were observed in the pathologic samples, as indicated by RT-PCR (fig. 3D), being about 10-times more compared to healthy. DSE expression seemed to be controlled by methylation of the promoter region in certain samples; the pathologic samples were hypomethylated compared to the macroscopically normal (fig. 4B).

## Discussion

We have studied the various chondroitin/dermatan polymerizing and modifying enzymes in healthy, macroscopically normal and cancerous human laryngeal cartilage. In previous studies [1], it was indicated that in the cartilaginous parts the chondroitin/dermatan profile is intensively altered in the advanced stages of cancer compared to the healthy cartilage. Therefore, we examined the chondroitin/dermatan polymerizing and modifying enzymes as the major etiology of these alterations. The extractability of the studied enzymes in PBS indicated that a major part secreted from the cells. In previous studies, the presence of such enzymes (CHSY1, D4ST1, C4ST1) in the conditioned medium in cultured cells was also identified [15].

The healthy laryngeal cartilage possesses great amounts of chondroitin/dermatan sulfate which in cancer are decreased [1], but this was not the same for the chondroitin synthesizing enzymes. Moreover, their alterations were not similar for all enzymes studied. CHSY3 and CHSY2 were decreased (fig. 1B, C and fig. 2A, C), whereas CHSY1 and CSGlcAT were increased (fig. 1A, D and fig. 2A). Since the decrease of the former was very high compared to the increase of the latter, the general decrease of CS/DS is explained.

The ratio of dermatan sulfate to chondroitin sulfate is increased in cancer [2] and thus the relative increase of dermatan sulfate should be attributed to DSE increased expression observed in the present work. Dermatan sulfate is considered as a glycosaminoglycan acting as a tumor suppressor and thus DSE may be considered as a tumor suppressor gene. Simultaneously, the D4ST1 decrease in cancer compared to healthy tissues may lead to more aggressive cancers due to the increased chondroitin formation. It is noteworthy that D4ST1 is pivotal for iduronic acid formation [16].

Chondroitin polymerizing factor and chondroitin synthase III were decreased in cancer compared to healthy tissues. In contrast, chondroitin synthase I is expressed mainly in cancer. These results further explain the obtained sulfation profile because CSA and mainly CSB (the C-4 sulfated chondroitin and dermatan sulfate, respectively) are synthesized preferentially by CHSY1. Therefore, the C-4 sulfation is favored in cancer. CHPF preferentially synthesizes C-6 chondroitin sulfate. It is noteworthy that all chondroitin synthases prefer non-sulfated and CSC substrates [6,9]. However, it has been shown that chondroitin polymerization is achieved by formation of complexes between the various chondroitin synthases and CSGlcA-T [17].

C-6 sulfation is decreased dramatically by stage whereas C-4 sulfation gradually. According to our study CHST3 gene expression was decreased in cancer compared to healthy tissues profoundly leading to C-6 sulfation decrease. D4ST1 gene expression was decreased, whereas C4ST1 gene was expressed more in the cancerous parts than in the macroscopically normal. Therefore, C-4 sulfation in CS-disaccharides could be relatively augmented in cancer but C-4 sulfation in DS-disaccharides decreased, yielding to an overall gradual C-4 sulfation decrease. Moreover, C4ST1 augmentation could lead to reduced C-6 sulfation in an indirect manner.

Data from other studies indicated that an additional glycosaminoglycan-modifying enzyme was found to have altered expression in cancer. D-glucuronyl C5 epimerase, acting exclusively on heparin and heparan sulfate, was found to be decreased in breast cancer [18]. On the other hand, dermatan sulfate epimerase (DSE), according to our and other studies [12], was increased. These findings suggest that both epimerases are affected in cancer leading to altered proteoglycan (both CS/DS and HS containing) biosynthesis.

The study of the glycosaminoglycan structure of the various proteoglycans, which is completed by the action of the studied enzymes, is important because it will lead to the deep comprehension of their role in cancer. Moreover, the examination of the regulatory mechanisms of these genes will guide to the exact understanding of the various pathways associated with cancer and thus will contribute to more targeted and effective treatment.

## Conclusions

The differential modification of the various glycosaminoglycans during cancer reflected differential expression of the enzymes involved in their biosynthesis. In our study, the most clear observations in laryngeal cancer were the significant decrease of CHSY3, CHST3 and D4ST1, and the significant increase of DSE. DSE is responsible for the epimerization of glucuronic acid in dermatan sulfate chains, which in addition require D4ST1 for their sulfation. The differential expression of only these two enzymes, which are highly responsible for the biosynthesis of dermatan sulfate, a glycosaminoglycan with tumor-inhibitory activity, indicates that a simple imbalance in enzymes' expression may affect tumor progression.

## Abbreviations

CHSY1: chondroitin synthase I; CHPF: chondroitin polymerizing factor; CHSY3: chondroitin synthase III; CSA: chondroitin sulfate A; CSB: chondroitin sulfate B; GAPDH: glyceraldehyde-3-phosphate dehydrogenase; CSGlcA-T: chondroitin sulfate glucuronyltransferase; CHST3: chondroitin 6-sulfotransferase; C4ST1: chondroitin 4-sulfotransferase; D4ST1: dermatan 4-sulfotransferase; DSE: dermatan sulfate epimerase: MSP; methylation specific PCR.

## Competing interests

The authors declare that they have no competing interests.

## Authors' contributions

DK, IET, GT, DHV and NSM carried out RT-PCR analysis, western blotting and data analysis, participated in the study design and drafted the manuscript. NSM, TAP, GT and PDG participated in the collection and characterization of patients' samples. All authors read and approved the final manuscript.
